# Detection of pathogenic copy number variants in children with idiopathic intellectual disability using 500 K SNP array genomic hybridization

**DOI:** 10.1186/1471-2164-10-526

**Published:** 2009-11-16

**Authors:** JM Friedman, Shelin Adam, Laura Arbour, Linlea Armstrong, Agnes Baross, Patricia Birch, Cornelius Boerkoel, Susanna Chan, David Chai, Allen D Delaney, Stephane Flibotte, William T Gibson, Sylvie Langlois, Emmanuelle Lemyre, H Irene Li, Patrick MacLeod, Joan Mathers, Jacques L Michaud, Barbara C McGillivray, Millan S Patel, Hong Qian, Guy A Rouleau, Margot I Van Allen, Siu-Li Yong, Farah R Zahir, Patrice Eydoux, Marco A Marra

**Affiliations:** 1 Department of Medical Genetics, University of British Columbia, Vancouver, Canada; 2 Child & Family Research Institute, Vancouver, British Columbia, Canada; 3 Victoria General Hospital, Victoria, British Columbia, Canada; 4 Children's & Women's Health Centre, Vancouver, British Columbia, Canada; 5 Genome Sciences Centre, British Columbia Cancer Agency, Vancouver, Canada; 6 CHU Sainte-Justine Research Center, Montréal, Québec, Canada; 7 Center for Excellence in Neuromics of Université de Montréal, CHU Sainte-Justine Research Center, Montréal, Québec, Canada

## Abstract

**Background:**

Array genomic hybridization is being used clinically to detect pathogenic copy number variants in children with intellectual disability and other birth defects. However, there is no agreement regarding the kind of array, the distribution of probes across the genome, or the resolution that is most appropriate for clinical use.

**Results:**

We performed 500 K Affymetrix GeneChip^® ^array genomic hybridization in 100 idiopathic intellectual disability trios, each comprised of a child with intellectual disability of unknown cause and both unaffected parents. We found pathogenic genomic imbalance in 16 of these 100 individuals with idiopathic intellectual disability. In comparison, we had found pathogenic genomic imbalance in 11 of 100 children with idiopathic intellectual disability in a previous cohort who had been studied by 100 K GeneChip^® ^array genomic hybridization. Among 54 intellectual disability trios selected from the previous cohort who were re-tested with 500 K GeneChip^® ^array genomic hybridization, we identified all 10 previously-detected pathogenic genomic alterations and at least one additional pathogenic copy number variant that had not been detected with 100 K GeneChip^® ^array genomic hybridization. Many benign copy number variants, including one that was *de novo*, were also detected with 500 K array genomic hybridization, but it was possible to distinguish the benign and pathogenic copy number variants with confidence in all but 3 (1.9%) of the 154 intellectual disability trios studied.

**Conclusion:**

Affymetrix GeneChip^® ^500 K array genomic hybridization detected pathogenic genomic imbalance in 10 of 10 patients with idiopathic developmental disability in whom 100 K GeneChip^® ^array genomic hybridization had found genomic imbalance, 1 of 44 patients in whom 100 K GeneChip^® ^array genomic hybridization had found no abnormality, and 16 of 100 patients who had not previously been tested. Effective clinical interpretation of these studies requires considerable skill and experience.

## Background

Chromosomal imbalance has been recognized as the most frequent cause of intellectual disability (ID) for 50 years [1-3]. Until recently, most of this genomic imbalance was diagnosed by cytogenetic analysis, but studies over the past few years have found that ID is caused by constitutional gains or losses of submicroscopic genomic segments even more often than by microscopically-apparent chromosomal aberrations [4-6]. Our ability to recognize these submicroscopic genomic changes, which are usually called copy number variants (CNVs), as the most frequent cause of ID depends on the use of Array Genomic Hybridization (AGH) (also known as array-comparative genomic hybridization, chromosomal microarray analysis, or copy number analysis). AGH can survey the entire genome for imbalance that is 1/100th the size of that detectable by conventional cytogenetic analysis.

Although AGH is now being used routinely as a clinical test for the identification of chromosomal imbalance in people with ID and other birth defects, controversy still exists regarding the most appropriate platform to use for this purpose. Initial clinical studies were done with arrays having a few thousand BACs distributed at 1-3 Mb intervals across the genome or with BACs targeted to regions where pathogenic submicroscopic deletions or duplications were known to occur. More recent studies have shown that arrays with higher resolution and genome-wide coverage provide better detection rates for pathogenic CNVs in children with ID and normal cytogenetic analysis [7-12]. Other methods have identified pathogenic CNVs that are too small to have been detected by the array platforms used in most AGH studies [13-15], so analysis at even higher resolution may be necessary to detect all pathogenic CNVs in children with ID.

Unfortunately, use of higher resolution AGH for detection of genomic imbalance is confounded because most CNVs are not pathogenic. Estimates of the mean number of apparently benign CNVs per person range from 6-824 in various studies, depending on the technology used to identify the variants and the size range used to define a CNV [16-25]. Sequencing of the complete diploid genomes of normal individuals has shown that the number of CNVs per person is actually even greater [26-29]. Distinguishing these benign CNVs from those that cause ID and other birth defects is the most serious challenge to routine clinical use of AGH.

We previously reported our findings using 100 K Affymetrix GeneChip^® ^AGH [10,16] to perform a genome-wide survey of benign and pathogenic CNVs in 100 idiopathic ID trios, each comprised of an affected child and both unaffected parents. Here we describe the results of a study of 100 new idiopathic ID trios, as well as 54 of the trios tested previously, using 500 K Affymetrix GeneChip^® ^AGH. We found that higher resolution AGH detected a larger number of apparently pathogenic CNVs in both groups. Many benign CNVs, including at least one that was *de novo*, were also detected with the 500 K AGH, but it was possible to distinguish benign and pathogenic CNVs with confidence in almost all cases.

## Results

We performed AGH with 500 K Affymetrix GeneChip^® ^arrays on 154 children with idiopathic ID and on both parents of each affected child. Fifty-four of these trios (called "the 100 K cohort") had previously been studied with lower-resolution 100 K GeneChip^® ^AGH [10,16]; the other 100 trios (called "the new cohort") were studied by AGH for the first time with the 500 K GeneChip^® ^arrays. Data were analyzed to determine copy number along the length of all chromosomes except the Y (for which there are no probes on either the 100 K or 500 K GeneChip^® ^arrays), and the findings for each child were compared with those for his or her parents. Autosomal CNVs seen in the child and in at least one parent were considered likely to be benign polymorphisms. Autosomal CNVs found in the child but not in either parent were evaluated by an independent method to confirm the presence of the CNV and its *de novo *occurrence. CNV calls on the X-chromosome in a female child were validated by an independent method if they appeared to have occurred *de novo; *CNV calls on the X-chromosome in a male child were validated by an independent method whether they appeared to be *de novo *or to have been inherited from the mother.

We found a total of 4577 hits (putative CNVs including at least 10 contiguous SNPs called by the SMD software with a p-value below 1 × 10^-8^) in the 462 samples (154 trios) analyzed by 500 K GeneChip^® ^AGH. This is an average of about 10 hits per sample, which is an underestimate of the total number of benign CNVs present because of the stringent cutoff used to obtain a false discovery rate of less than 5% (see Methods).

Within the 54 trios who were studied with both platforms, we found four times as many hits with 500 K AGH as we did with 100 K AGH (Table 1). The ratio of the number of putative CNVs called with the 500 K platform to the number called with the 100 K platform was 11.0 for CNVs between 100 kb and 200 kb in size but was lower for both smaller and larger CNVs (4.1 for those < 100 kb and 3.4 for those between 200 kb and 500 kb).

**Table 1:** Putative CNVs called on 100 K and 500 K AGH in 54 trios tested with both technologies.

	Comparison	Child to Father		Child to Mother	
	
	Array	100 K	500 K	100 K	500 K
**All CNV Calls**	Number	125	497	110	501
	
	Number per Child	2.31	9.20	2.04	9.28
	
	Statistical Significance	t = 7.18, p < 0.0001		t = 6.67, p < 0.0001	

**CNV Calls < 100 kb**	Number	59	237	53	221
	
	Number per Child	1.06	4.39	0.98	4.09
	
	Statistical Significance	t = 7.82, p < 0.0001		t = 5.19, p < 0.0001	

**CNV Calls ≥ 100 kb and < 200 kb**	Number	13	130	12	146
	
	Number per Child	0.24	2.41	0.22	2.70
	
	Statistical Significance	t = 5.39, p < 0.0001		t = 6.27, p < 0.0001	

**CNV Calls ≥ 200 kb and < 500 kb**	Number	33	101	25	98
	
	Number per Child	0.61	1.87	0.46	1.81
	
	Statistical Significance	t = 4.66, p < 0.0001		t = 5.25, p < 0.0001	

Two-tailed statistical significance was calculated with Student's t-test.

The 4577 putative CNVs called in all 154 trios were subjected to further bioinformatic analysis (see Methods) to produce a final annotated list of 58 apparently *de novo *CNVs and two cases of mosaic trisomy that were called in 50 patients. These apparent genomic imbalances were subjected to validation by independent methods. Thus, an apparent *de novo *CNV call that required independent validation was made in about 1 child in 3. Thirty-three of these CNVs in 30 patients and both cases of mosaic trisomy were confirmed to be *de novo *by an independent method and are described in detail below. The other putative CNVs were found to be present in both of the parents as well as in the child (false negative AGH calls in the parents) in two instances or could not be confirmed to be present in the child (false positive AGH calls) in 22 instances. Altogether, false positive CNV calls were made in 21 (13.6%) of the 154 trios studied and false negative CNV calls were made in 2 (1.3%) of the 154 trios studied. In one other trio (Family 5202), an apparent *de novo *deletion of chromosome 14 called on AGH in the child was found by FISH to be a duplication of the region in both parents instead.

Nineteen of 100 children with ID in the new cohort were found by Affymetrix 500 K GeneChip^® ^AGH to have *de novo *genomic imbalance that was confirmed by FISH, MLPA, AGH on an Agilent^® ^244 K platform or cytogenetic re-analysis (Table 2). One of these children (Patient 8056) had mosaic trisomy 9, and two were found to have *de novo *unbalanced reciprocal translocations - a der(10)t(2;10)(q37;q26.13) in Patient 873 and a der(4)t(4;8)(p16.1;p23.1) in Patient 5814 - each producing both a terminal duplication and a terminal deletion identified by AGH. We found *de novo *submicroscopic deletions in 13 other patients and *de novo *submicroscopic duplications in three other patients. The deletions ranged in size from 89 kb to 11.0 Mb; six were less than 1 Mb. The duplications ranged in size from 362 kb to 11.1 Mb; one was less than 1 Mb.

**Table 2:** Genomic imbalance and uniparental disomy detected by 500K GeneChip^® ^AGH in the new cohort.

Patient	Change	Location	SNP Count	Start → End	Size (bp)	Validation	Phenotype	RefSeq Genes Involved*	Comments	Interpretation
9133	Deletion	1p36.32-p36.33	198	769,185 **→ ** 3,581,308	2,812,123	FISH, MLPA	9 year-old female with obesity, moderate cognitive impairment, myoclonus, polyphagia, hypotonia, narrow frontal area, deep-set eyes, prominent orbital rims, short nose with low nasal bridge and upturned nasal tip, midface retrusion, short philtrum, tented upper lip, thoracic kyphosis, small distal phalanges of the toes, strabismus, and 11 ribs	~70 genes including *AGRN, GNB1, PEX10, PRKCZ, SKI*, and *TP73*	This CNV is included in the 1p36 deletion syndrome critical region, and the patient's clinical features are compatible with that syndrome [73].	Pathogenic

873	Duplication^†^	2q37	1801	231,577,285 **→ ** 242,663,303	11,086,018	FISH, MLPA	15 year-old male with severe cognitive impairment, birth weight < 1^st ^centile, birth length < 1^st ^centile, head circumference at birth < < 10^th ^centile, hypotonia, microcephaly, contractures of hips and knees, hypoplastic scrotum, undescended testes, prominent, cup-shaped ears, narrow bifrontal diameter, broad nasal root, prominent epicanthal folds, bilateral clinodactyly V, ataxic gait, progressive joint contractures and muscle wasting of lower extremities, mixed hearing loss and hypoplastic inferior cerebellar vermis, partial dysgenesis of corpus callosum, and narrow pons and brain stem on MRI	~100 genes including *AGXT, ATG16L1, CAPN10, CHRND, CHRNG, COL6A3, D2HGDH, GBX2, HDAC4, MLPH, PDCD1, PER2, SAG *and *UGT1A1*		Pathogenic
					
	Deletion^†^	10q26.13	1920	126,415,527 **→ ** 134,032,911	7,617,384			~75 genes including *ADAM12, CTBP2, DOCK1, DPYSL4, FGFR2, OAT*, and *UROS*		

6473	Deletion	4p16.3	337	190,631 **→ ** 3,277,436	3,086,805	Cytogenetic re-analysis, FISH	3 year-old male with fetal growth retardation, length 2 standard deviations below the mean for age, weight 3-4 standard deviations below the mean, head circumference 3-4 standard deviations below the mean, global developmental delay, seizures, triangular face, small jaw, posteriorly rotated ears, 2° hypospadias, and ataxia	~45 genes including *ADD1, FGFR3, HTT, IDUA, LETM1, PDE6B, SH3BP2*, and *WHSC1*	The deletion in this patient includes the Wolf- Hirschhorn syndrome critical region, and the clinical features are compatible with that syndrome [74].	Pathogenic

5814	Deletion^†^	4p16.1-p16.3	1520	13,255 **→ ** 8,472,657	8,459,402	Cytogenetic re-analysis, MLPA	13 month-old female with length below the 3^rd ^centile, microcephaly, developmental delay, bilateral preauricular pits, and submucus cleft palate	~90 genes including *ADD1, ADRA2C, CRMP1, EVC*	The deletion includes the Wolf- Hirschorn syndrome critical region.	Pathogenic
					
	Duplication^†^	8p23.1-p23.3	2579	180,568 **→ ** 6,898,076	6,717,508			20 genes including *ARHGEF10, CLN8, DLGAP2*, and *MCPH1*		

216	Deletion	6p21.33	42	29,937,087 **→ ** 30,026,517	89,430	MLPA	18 year-old male with postnatal onset growth retardation, unilateral sensorineural deafness, narrow face, bulbous nasal tip and mild intellectual disability.	HCG9, MICD and 5 pseudogenes	Deletion is homozygous in child, heterozygous in both parents (not *de novo *mutation)	Not pathogenic for ID

3160	Deletion	6 q21-q22.31	1596	111,979,175 **→ ** 121,506,916	9,527,741	FISH	8 year-old female with moderate developmental delay, hypotonia, microcephaly, brachycephaly, epicanthic folds, small ears with hypoplastic lobes, and micrognathia	~40 genes including *ASF1A, COL10A1, FRK, FYN, GOPC, HDAC2, LAMA4, MCM9, PLN, TSPYL1*, and *WISP3*		Pathogenic

2200	Deletion	7p15.3	2320	14,141,506 **→ ** 24,950,414	10,808,908	FISH	11 1/2 year-old female with head circumference at the 2^nd ^centile, mild cognitive impairment, sensorineural hearing loss, cleft palate, craniosynostosis, unilateral ptosis and esotropia, orbital rim hypoplasia, malar and midface hypoplasia, low-set ears with incomplete out-folding of superior helix, brachydactyly and syndactyly of digits, broad thumbs, decreased range of motion in elbows, and leg length discrepancy	~40 genes including *DFNA5, DGKB, DNAH11, FAM126A, HDAC9, IL6*, and *RAPGEF5*		Pathogenic

9938	Deletion	7q22.1	170	98,211,585 **→ ** 100,553,755	2,342,170	FISH	14 year-old female with height < 5^th ^centile, weight < 5^th ^centile, head circumference < 5^th ^centile, severe cognitive impairment, left sensorineural hearing loss, close-set eyes, broad nasal root, marked retrognathia, high-arched palate, small and narrow feet, short 2-5th toes with hypoplastic nails, atrio-vetricular septal defect, and polyarticular arthritis	~70 genes including *ACHE, ACTL6B, CYP3A5, EPO, MUC3A, SERPINE1, SMURF1*, and *TFR2*		Pathogenic

1594	Duplication	8 q12	1220	58,388,614 **→ ** 65,306,097	6,917,483	FISH	1 1/2 year-old female with height > 97^th ^centile, head circumference at 2^nd^-5^th ^centile, developmental delay, Duane anomaly, broad glabella, epicanthic folds with telecanthus, upslanting palpebral fissures, pre-auricular pits, large ears, atrial and ventricular septal defects, and renal reflux	15 genes including *ASPH, CHD7, RAB2A, RLBP1L1, TOX*, and *TTPA*		Pathogenic

663	Deletion	9 p13.3	800	34,144,847 **→ ** 38,736,451	4,591,604	FISH	5 1/2 year-old female with height at the 5^th ^centile, developmental delay, tremor, ocular hypertelorism, epicathal folds, double hair whorl, bilateral ptosis, short upturned nose, flattened philtrum, underdeveloped genitalia, and pigmentary retinal changes	~75 genes including *CNTFR, DNAI1, DNAJB5, FANCG, GALT, GBA2, GNE, GRHPR, NPR2, PAX5, RECK, SHB, TPM2, UNC13B*, and *VCP*	The deleted region in this patient is completely included in the region deleted in patient 9346.	Pathogenic

9346	Deletion	9p11.2-p13.3	880	33,702,471 **→ ** 44,744,675	11,042,204	FISH	9 1/2 year-old female with moderate cognitive impairment, seizures, tremor, cataract, broad frontal area with bossing, arched eyebrows, low nasal bridge, and short, upturned nose	~85 genes including *CNTFR, DNAI1, DNAJB5, FANCG, GALT, GBA2, GNE, GRHPR, NPR2, PAX5, RECK, SHB, TPM2, UNC13B*, and *VCP*	The deleted region in this patient includes the entire segment deleted in Patient 663.	Pathogenic

523	Deletion	9q34.3	36	139,516,033 **→ ** 139,814,485	298,452	FISH	4 year-old female with moderate developmental delay, hypotonia, microcephaly, flat face with upslanting palpebral fissures, ocular hypotelorism, synophrys, and anteverted nares	7 genes including *EHMT1*	This deletion is within the critical region for the 9q subtelomeric deletion syndrome[75], and the child's clinical features are compatible with that syndrome.	Pathogenic

8056	Mosaic Trisomy	9	27,641	whole chromosome		FISH	2 1/2 year-old male with weight < 5^th ^centile, developmental delay, preauricular skin tags, hypospadias, and cryptorchidism	Numerous	Clinical features compatible with mosaic trisomy 9 syndrome[76,77]	Pathogenic

6904	Uniparental Disomy	11p11.2-pter	See Table 3	196,767 **→ ** 44,589,530	44,392,763	Micro-satellite markers	11 year-old female with height < 5^th ^centile, gross and fine motor delay, hypotonia, and moderate mental handicap	Numerous	Mosaic paternal isodisomy; phenotype not compatible with Beckwith-Wiedemann syndrome	Uncertain

9897	Deletion	13q33.3-q34	530	107,271,189 **→ ** 109,368,996	2,097,807	FISH	10 year-old female with fetal growth retardation, moderate cognitive impairment, upslanting palpebral fissures, and retrognathia	6 genes including *IRS2, LIG4*, and *MYO16*		Pathogenic

818	Deletion	14q11.2	24	21,929,710 **→ ** >22,036,502	106,792	Fosmid FISH (variable)^§^	6 1/2 year-old male with weight < 5^th ^centile, height at 5^th ^centile, mild cognitive impairment with particular delay in language, mild mid-face hypoplasia with narrow high-arched palate, mild micrognathia, pre-auricular pit, joint laxity, bilateral clinodactyly of hands, and bilateral 2-3 syndactyly of toes	Multiple T-cell receptor alpha-chain V, J, and region genes	Highly polymorphic region	Not pathogenic for ID

1658	Uniparental Disomy	16	See Table 3	Whole chromosome	Whole chromosome	Micro-satellite markers	5 year-old female with normal growth, severe mental handicap, seizures, self-abusive behaviour, deep and dark creases under the eyes, mild mid-face hypoplasia, and large mouth	Numerous		Uncertain

2106	Deletion	17q21.31	149	41,049,321 **→ ** 41,564,451	515,130	FISH	15 year-old male with fetal growth retardation, mild cognitive impairment, attention deficit disorder, sagittal craniosynostosis, long face with malar hypoplasia and mild rectrognathia, short and upslanting palpebral fissures, low-set ears, unilateral cryptorchidism, partial agenesis of the corpus callosum, and dilatation of the aortic root	5 genes including *MAPT*	The deleted segment includes the critical region for the 17q21.31 deletion syndrome, [78] and this patient's clinical features are compatible with that syndrome.	Pathogenic

8619	Deletion	21q22.11	13	33,902,218 **→ ** 34,087,893	185,675	Agilent 244 K AGH	24 year-old male with prenatal and postnatal growth retardation, moderate to severe intellectual disability, severe hypotonia, microcephaly, metopic craniosynostosis, cleft palate, down-slanting palpebral fissures, low-set ears, wide nasal base, retrognathia, tetralogy of Fallot, cryptorchidism, and joint hyperextensibility	5 genes including *SYNJ1*	No polymorphisms of region reported	Uncertain
					
	Deletion	22q11.2	110	19,062,809 **→ ** 19,785,125	722,316	FISH		14 genes including *BCR, DGCR8, HIRA, MAPK1, PRODH, SNAP29, SEPT5, SERPIND1*, and *TBX1*	The deleted segment is included in the 22q11 deletion syndrome critical region, and the phenotype is compatible with other reported cases of distal 22q11.2 microdeletion [79-81]	Pathogenic

9979	Duplication	22q11.21	57	19,429,297 **→ ** 19,791,607	362,310	FISH	20 year-old female with short stature, mild mental deficiency, cleft palate, and micrognathia	9 genes including *PI4KA SERPIND1, LZTR1, SNAP29*	Polymorphic region	Uncertain

1128	Duplication	Xq12-q21.1	475	67,088,023 **→ ** 76,204,344	9,116,321	FISH	11 year-old male with normal growth, severe developmental delay, hypotonia, brachycephaly, bilateral epicanthic folds, and posteriorly rotated ears with hypoplastic helices	~60 genes including *ABCB7, DLG3, EDA, EFNB1, GJB1, IGBP1, IL2RG, OPHN1* *NAP1L2, NLGN3, PHKA1, SLC16A2, TAF1*, and *ZDHHC15*		Pathogenic

The table includes all *de novo *CNVs, mosaic trisomy and UPD detected by 500 K AGH and confirmed by an independent method in 100 children with idiopathic ID. Breakpoints are shown on Human Genome Assembly Build 36.1.

* The approximate number of RefSeq genes for each CNV is given, but only the most likely genes for the phenotype are named.

^† ^Unbalanced reciprocal translocation.

^§ ^Interphase FISH in patient 818 showed some cells with no signals, some with 1 signal and some with two signals for a probe in the region of the deletion detected by AGH. This was interpreted as evidence of somatic mosaicism for this deletion.

**Figure 1:**
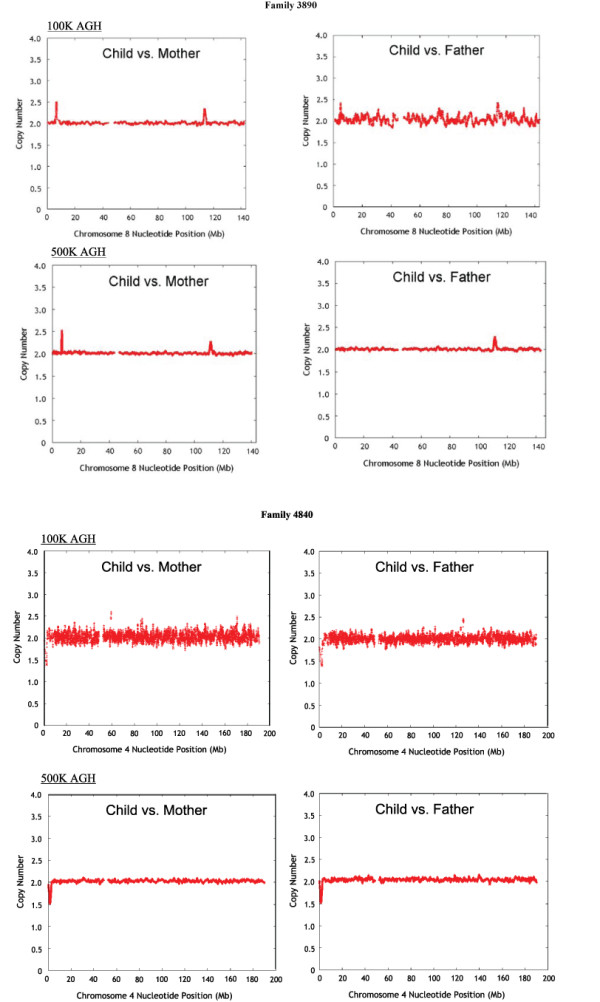
***De novo *CNVs detected with 500 K but not 100 K AGH in children with idiopathic ID**. The plots show *in silico *comparison of estimated copy number in child versus mother (left) and child versus father (right) at each position along the chromosome. *Upper panel*: Smoothed copy number plots for chromosome 8 in Family 3890. Affymetrix 100 K AGH is shown with a 59 SNP window, and Affymetrix 500 K AGH is shown with a 170 SNP window. Note duplication at 111,442,951 to 113,003,770 bp that is apparent on 500 K AGH but was not called on our original analysis of the 100 K AGH data. The CNV is represented by 59 SNPs on the 100 K array. *Lower panel*: Smoothed copy number plot for chromosome 4 in Family 4840. Affymetrix 100 K AGH is shown with a 13 SNP window, and Affymetrix 500 K AGH is shown with a 145 SNP window. Note deletion at 1,346,924 to 2,846,261 bp that is apparent on 500 K AGH but was not called as *de novo *by 100 K AGH on our initial analysis. This CNV is represented by 17 SNPs on the 100 K array.

Using 500 K Affymetrix GeneChip^® ^AGH, we also confirmed the genomic imbalance that had previously been identified in 10 of the 54 ID trios from the 100 K cohort (Table 3 and Additional File 1: Supplemental Table S1). In addition, we identified and confirmed by FISH two *de novo *CNVs that were not called on the 100 K assay - a 1.6 Mb duplication of 8q23.2-23.3 in Patient 3890 and a 1.5 Mb deletion of 4p16.3 in Patient 4840 (Table 3 and Figure 1).

**Table 3:** Genomic imbalance detected by 500K AGH in 54 idiopathic ID trios from the 100K cohort.

	Affymetrix 100K AGH				Affymetrix 500K AGH			
Family	Change	Location	Start → End	Size (bp)	Change	Location	Start → End	Size (bp)
1895	Deletion	13q12.11-q12.13	18,867,056 **→ ** 24,517,730	5,650,674	Deletion	13q12.11-q12.12	18,876,037 **→ ** 24,330,232	5,454,195

3476	Deletion	4q21.21-q22.1	82,008,594 **→ ** 93,076,278	11,067,684	Deletion	4q21.21-q22.1	82,429,950 **→ ** 91,434,337	9,004,387

3890	Normal				Duplication	8q23.2-q23.3	111,442,951 **→ ** 113,003,770	1,560,819

4794	Duplication	16p13.3	925,718 **→ ** 3,864,938	2,939,220	Duplication	16p13.3	2,681,813 **→ ** 3,927,524	1,245,711

4818	Deletion	12q14.2-q15	63,342,649 **→ ** 66,780,095	3,437,446	Deletion	12q14.2-q15	63,362,084 **→ ** 66,737,699	3,375,615

4840	Normal				Deletion	4p16.3	1,346,924 **→ ** 2,846,261	1,499,337

5003	Deletion	2p16.3	50,799,281 **→ ** >51,120,644	321,363	Deletion	2p16.3	50,829,675 **→ ** 51,120,302	290,627

5566	Deletion	14q11.2	20,741,117 **→ ** 20,918,741	177,624	Deletion	14q11.2	20,787,740 **→ ** 20,988,716	200,976

5994	Mosaic Trisomy 9	Whole Chromosome			Mosaic Trisomy 9	Whole Chromosome		

6545	Deletion	7p22.2-p22.1	3,498,135 **→ ** 7,134,218	3,636,083	Deletion	7p22.2-p22.1	3,657,805 **→ ** 6,165,597	2,507,792

7807	Deletion	22q12.1	26,144,210 **→ ** 27,557,971	1,413,761	Deletion	22q12.1	26,293,416 **→ ** 27,462,458	1,169,042

8326	Deletion	14q11.2	19,584,863 **→ ** 21,207,935	1,623,072	Deletion	14q11.2	19,592,409 **→ ** 21,256,822	1,664,413

The table includes all de novo CNVs and mosaic trisomy detected by 500 K AGH and confirmed by an independent method in a selected group of 54 trios who had previously been tested by 100 K GeneChip^® ^AGH [10]. Breakpoints are shown on Human Genome Assembly Build 36.1.

**Figure 2:**
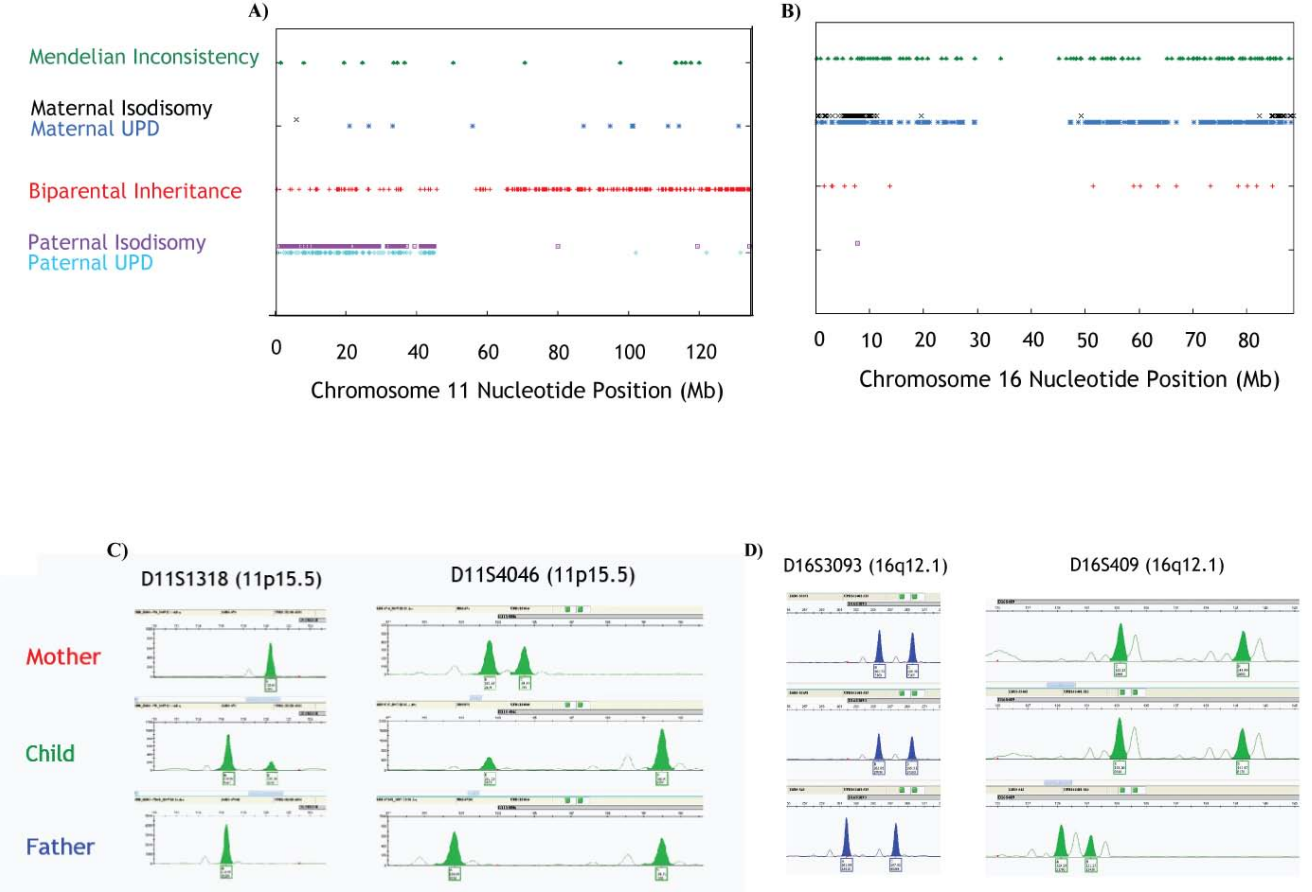
**Uniparental disomy detected with Affymetrix 500 K AGH in two patients with idiopathic ID**. **A) **The child in Family 6904 was found to have mosaic paternal uniparental disomy, probably isodisomy, of chromosome 11 p15.5-p11.2. SNP genotypes obtained by Affymetrix 500 K AGH and interpreted for the trio as described in the Methods are shown along the length of chromosome 11. **B) **The child in Family 1658 was found to have maternal uniparental disomy for all of chromosome 16. The ends of both chromosome arms (proximal to 11,559,620 bp and distal to 84,641,383 bp) appear to be isodisomic; the central portion of the chromosome is heterodisomic. SNP genotypes obtained by Affymetrix 500 K AGH and interpreted for the trio as described in the Methods are shown along the length of chromosome 16. **C) **Allelic imbalance, compatible with paternal isodisomy and mosaicism, for two informative microsatellite markers in the involved region of chromosome 11 in the child in Family 6904. The location of each marker is shown in brackets. **D) **Maternal heterodisomy for two informative microsatellite markers in the involved region of chromosome 16 in the child in Family 1658. The location of each marker is shown in brackets.

We found two instances of uniparental disomy (UPD), diagnosed by the occurrence of mendelian inconsistency in a region with a normal copy number of 2 [30], among the 100 ID trios in the new cohort studied by 500 K Affymetrix GeneChip^® ^AGH (Table 4 and Figures 2A and 2B). Patient 6904 has mosaic paternal isodisomy of most of the short arm of chromosome 11. Patient 1658 has maternal UPD 16, being heterodisomic for the central portion of the chromosome and isodisomic for both ends. Both cases were confirmed to be disomic with microsatellite markers (Figures 2C and 2D).

**Table 4:** UPD detected in 100 children with idiopathic ID from the new cohort.

Patient	Chromosome	Affected Region			SNPs in Region of UPD					Interpretation
			
		Start	End	Size (bp)	Total	Paternal UPD	Maternal UPD	BPI*	MI*	
6904	11	196,767	44,589,530	44,392,763	9,857	h = 192* i = 456*	h = 89 i = 1	59	8	Paternal Isodisomy

1658	16	14,139	11,559,620	11,545,481	2,837	h = 0 i = 1	h = 193 i = 188	5	19	Maternal Isodisomy
		
		11,559,620	84,641,380	73,081,760	11,698	h = 0 i = 0	h = 638 i = 3	12	95	Maternal Heterodisomy
		
		84,641,383	88,668,856	4,027,473	772	h = 0 i = 0	h = 25 i = 56	1	7	Maternal Isodisomy

The table includes all UPD detected by 500 K GeneChip^® ^AGH and confirmed by an independent method in 100 children with idiopathic ID. All mendelian inconsistencies observed were single. SNPs that are not listed as paternal UPD, maternal UPD, BPI* or MI* were uninformative with respect to UPD. Breakpoints are shown on Human Genome Assembly Build 36.1.

* Abbreviations used: h = heterodisomy, i = isodisomy, BPI = biparental inheritance, MI = mendelian inconsistency.

We judged the mosaic trisomy 9, both unbalanced reciprocal translocations, 11 of the other *de novo *deletions, and two of the other *de novo *duplications found in the new cohort to be pathogenic (Table 2). A mosaic 107 kb *de novo *deletion of chromosome 14 q11.2 (Patient 818) and a homozygous 89 kb deletion of the HLA-G region that resulted from transmission by parents who both carried heterozygous deletions of the same region (Patient 216) were judged to be benign variants. A 186 kb deletion of chromosome 21q22.11 (Patient 8619), a 362 kb duplication of chromosome 22 q11.21 (Patient 9979) and both cases of UPD (Patients 1658 and 6904) were of uncertain clinical significance.

Of the two *de novo *CNVs identified by 500 K Affymetrix GeneChip^® ^AGH in the 44 children with ID whose 100 K GeneChip^® ^AGH studies had been interpreted as normal (Table 3), one is likely to be pathogenic and the other is of uncertain clinical significance. A 1.5 Mb deletion of 4p16.3 in Patient 4840 is pathogenic because of its size, the inclusion of two genes that have been implicated in the Wolf-Hirschhorn syndrome (*WHSC1 *and *WHSC2*), overlap with known pathogenic CNVs, and a compatible clinical phenotype. The 1.6 Mb duplication of 8q23.2-23.3 in Patient 3890 is of unknown clinical significance. It includes no RefSeq genes but involves a large (1.6 Mb) genomic region, most of which has never been reported to be polymorphic in normal individuals.

## Discussion

Because its detection rate for pathogenic genomic imbalance is much higher than that of conventional cytogenetic analysis, a consensus has developed that AGH should be used clinically for the evaluation of patients with ID and other birth defects [31-38]. It is clear that AGH using "targeted" arrays that only include probes for genomic regions known to be involved in microdeletion or microduplication syndromes has substantially lower detection rates for CNVs that cause ID than AGH using arrays that provide genome-wide coverage [37,39-41]. Beyond this, however, there is no agreement regarding the kind of array, the distribution of probes across the genome, or the resolution that is most appropriate for clinical use. Although BAC arrays were initially used, most clinical laboratories now prefer oligonucleotide arrays because high-quality platforms that produce consistent results are reliably available from commercial sources. In addition, the use of larger numbers of smaller probes on oligonucleotide arrays permits more precise delineation of the breakpoints of CNVs that are detected, which facilitates genotype-phenotype correlation and clinical interpretation. AGH with a SNP array provides the additional advantage of generating genotypes that can be used to verify family relationships and find uniparental disomy as well as a second method (in addition to hybridization intensity) for identifying genomic imbalance [10,42-44].

We previously reported that 100 K Affymetrix GeneChip^® ^AGH is a robust platform for the detection of pathogenic CNVs in patients with ID [10]. Here we show that the detection rate of CNVs among such patients is higher with 500 K GeneChip^® ^AGH than with 100 K GeneChip^® ^AGH. We made about four times as many CNV calls overall with the 500 K platform as with the 100 K platform when using the same method of bioinformatic analysis in 54 trios studied with both technologies (Table 1). We also found 18 instances of pathogenic genomic imbalance in 16 of 100 children with ID and normal cytogenetic analysis studied by 500 K GeneChip^® ^AGH (Table 2), compared to 11 instances of pathogenic genomic imbalance in 11 of 100 similarly-ascertained children tested by 100 K GeneChip^® ^AGH in our previous study. Although the higher detection rate we observed with the 500 K platform may have occurred by chance because we just happened to include a few more patients with such genomic changes in the new cohort than in the 100 K cohort, we also found two additional *de novo *CNVs by 500 K GeneChip^® ^AGH among the 44 children whose 100 K AGH was interpreted as normal in our earlier studies (Figure 1 and Table 3).

We detected three apparently *de novo *CNVs smaller than 200 kb among the 100 trios tested with 500 K GeneChip^® ^AGH in the new cohort (a 107 kb deletion of chromosome 14 in Patient 818, a 186 kb deletion of chromosome 21 in Patient 8619, and an 89 kb deletion of chromosome 6 in Patient 216 that was actually a homozygous loss inherited from two heterozygous parents), but none of these CNVs was clearly pathogenic. The overall size distribution of pathogenic CNVs detected by 500 K GeneChip^® ^AGH among 154 children with idiopathic ID in the present study is similar to that observed by 100 K GeneChip^® ^AGH among 100 children with idiopathic ID whom we studied previously [10] (see Additional File 2: Supplemental Figure S1). The higher detection rate on the 500 K array therefore appears to be related more to better probe coverage in relevant genomic regions and an improved ability to distinguish CNVs from background noise, rather than to a capacity to identify much smaller pathogenic CNVs. This is illustrated in Families 3890 and 4840 (Figure 1), in which a 1.6 Mb duplication of 8q23.2q23.3 and a 1.5 Mb deletion of 4p16.3, respectively, are obvious on the 500 K AGH but were not called on the 100 K analysis. In retrospect, the 4p16.3 deletion in Patient 4840 can be seen on the 100 K AGH copy number plot despite the noisy data, but it was not called by either the automated analysis or visual inspection of these plots when the initial study was done. Our failure to detect the *de novo *duplication of 8q23.2q23.3 in Patient 3890 was probably caused by the noisy data in the father's study.

Distinguishing benign CNVs from those that cause ID and other birth defects is a critical issue in routine clinical use of AGH. Benign CNVs occur in all people and are a major source of genetic variation in the normal population [21,27,29]. Most apparently benign CNVs over 2 kb in size occur as polymorphisms with minor allele frequencies of at least 5% [21] and are inherited from a parent [21,23,45,46].

Benign and pathogenic CNVs can usually be distinguished in patients with ID and other birth defects by inheritance and genotype-phenotype correlation [5,33,47]. In this study, we identified a mean of about 10 CNVs per subject in the 154 ID trios tested by 500 K AGH. The vast majority of these CNVs were characterized as benign because they were inherited from a normal parent. Genomic imbalance that occurs *de novo *in a patient with ID whose parents are normal is more likely to be pathogenic than genomic imbalance that was inherited unchanged from a normal parent. We performed AGH on both parents of every child with ID to determine the inheritance of the CNVs found in the child, but this is sometimes not possible in clinical practice. In such instances it is necessary to infer likely *de novo *occurrence by information obtained from populations that have previously been studied [19,22-24,48,49]. Great care must be taken to avoid misinterpretation when this is done, especially if the available data were obtained with lower resolution AGH, the phenotypic characteristics of the comparison population are uncertain, reported polymorphic CNVs do not have exactly the same breakpoints as the CNV of interest, or the population frequency of a previously-reported CNV is unknown.

Compelling evidence that a CNV in a person with ID is pathogenic exists if the genomic imbalance is known to cause the patient's phenotype in other individuals, e.g., if a child with del 9q34.3 has features of the 9q subtelomeric deletion syndrome (Patient 523), a child with del 1p36.32p36.33 has features of the 1p36 deletion syndrome (Patient 9133), or a child with del 17q21.31 has features of the syndrome associated with this deletion (Patient 2106). Pathogenicity is also supported when a CNV includes a gene that is known to cause the patient's phenotype when inactivated (if the CNV is a deletion) or over-expressed (if the CNV is a duplication). On the other hand, a CNV is unlikely to be pathogenic if it involves a highly polymorphic region in which genomic loss (or gain, whichever is present in the patient) of the entire involved segment is known to occur in normal people.

If a direct genotype-phenotype correlation of this kind cannot be made in a particular case, certain genetic features of the CNV may provide clues to its pathogenicity. CNVs that are larger and those that involve gene-rich regions are more likely to be pathogenic than CNVs that are smaller and involve only gene-poor regions [5,47]. In addition, clinical experience suggests that deletions are more likely to be pathogenic than duplications [47]. The genetic content of a CNV may also make pathogenicity more or less likely. For example, involvement of a gene that lies within a pathway that is known to contain other dosage-sensitive genes associated with a similar phenotype strengthens the possibility of pathogenicity, while a CNV that does not contain any genes that are expressed in relevant tissues during embryogenesis is unlikely to be pathogenic for ID.

There are, of course, exceptions to each of these "rules". Some benign CNVs arise *de novo *[21,23,45,46,50], as appears to have occurred in the *de novo *deletion of chromosome 14q11.2 we found in Patient 818. The 107 kb region involved is highly polymorphic and contains several T-cell receptor variable region genes. On the other hand, some CNVs that are inherited from a normal parent are pathogenic for ID. Examples include maternal transmission of a *UBE3A *deletion to a child with Angelman syndrome [51], maternal transmission of a *MECP2 *duplication to a son [52], and CNVs such as dup 22q11.2 [53,54] or del 1q21.2 [55] that can cause ID but exhibit incomplete penetrance.

Although large (> 250 kb) CNVs are often pathogenic, they may be benign [19,29]. Most benign CNVs are small (< 250 kb) [19,27,29], and it seems probable that the smaller a CNV, the more likely it is to be benign. Nevertheless, no clear size distinction exists between benign and pathogenic CNVs. We found pathogenic CNVs as small as the 298 kb deletion of 9q34.3 in Patient 523 in this study, and others have reported even smaller pathogenic CNVs [13-15,56-58]. Expression patterns, functional annotation and animal models can provide important clues to pathogenicity in some cases, but without knowledge of the phenotypic effects of a copy number alteration in humans, one can rarely, if ever, be certain whether a novel gain or loss of a particular genomic region can produce ID or other birth defects.

In our 500 K AGH study of 154 ID trios, 58 *de novo *CNVs were called by bioinformatic analysis, and 33 of these CNVs were confirmed and shown to be *de novo *by an independent method. Because we could assess the phenotypes of our patients in detail and correlate the findings with those obtained by AGH, we were able to determine with confidence whether the genomic imbalance we observed was pathogenic or not in every case studied except three (Tables 2 and 3). Such genotype-phenotype correlation is critical to determining the effects of novel CNVs detected by AGH in patients with ID.

A CNV of uncertain clinical significance was encountered in three (1.9%) of the 154 trios analyzed in this series - a 362 kb duplication of 22q11.21 in Patient 9979 (Table 2), a 186 kb deletion of 21q22.11 in Patient 8619 (Table 2) and a 1.6 Mb duplication of chromosome 8q23.2q23.3 in Patient 3890 (Table 3). This rate of CNVs of uncertain clinical significance is similar to that reported in large series of patients with ID and other birth defects studied by AGH with "targeted" chips [31,35,59].

We were uncertain of the clinical significance of either case of UPD that we detected. Although only a few liveborn children with UPD 16 have been recognized, the reported experience does not suggest that UPD 16 can cause the abnormalities observed in Patient 1658 [60,61]. Paternal UPD 11p15 can produce Beckwith-Wiedemann syndrome [62], but this phenotype is very different from that observed in the affected child in Family 6904. However, as both of these cases involved isodisomy of a portion of the chromosome, we cannot rule out the possibility that the abnormal phenotype was produced by homozygosity for a recessive mutant allele [63,64]. Although the detection of UPD in addition to alterations of copy number is a theoretical benefit of using an array that includes probes for SNPs, the clinical utility of genome-wide screening for UPD in patients with idiopathic ID and other birth defects is uncertain.

We detected more pathogenic CNVs with 500 K AGH than with 100 K AGH, but some CNVs that were present among our patients were not detected using the 500 K assay. For example, our 500 K GeneChip^® ^analysis failed to identify a pathogenic 83 kb deletion of chromosome 16p13.3 (3,862,993 bp to 3,945,522 bp) involving the *CREBBP *gene (Patient 5121). This *de novo *deletion was found by AGH on the Agilent^® ^244 K platform and was confirmed by MLPA. The patient is an 8 year-old boy whose clinical features are characteristic of the Rubinstein-Taybi syndrome, which has been associated with deletions and other mutations of *CREBBP *in other patients [65,66]. The 83 kb genomic region deleted in our patient is poorly represented on the 500 K GeneChip^® ^arrays, with a total of only 15 SNPs. SNP arrays have uneven genomic coverage, and the addition of non-polymorphic oligonucleotide probes to the design of arrays like the one used in this study has been shown to provide substantially better detection of CNVs [21].

## Conclusion

Affymetrix GeneChip^® ^500 K array genomic hybridization performed in individuals with idiopathic intellectual disability detected pathogenic genomic imbalance in 10 of 10 patients in whom 100 K GeneChip^® ^array genomic hybridization found genomic imbalance, 1 of 44 patients in whom 100 K GeneChip^® ^array genomic hybridization had found no abnormality, and 16 of 100 patients who had not previously been tested. Further improvements in array design, ongoing improvements in AGH software, and continuing enhancement of resources like DECIPHER https://decipher.sanger.ac.uk/ and the Toronto Database of Genomic Variants http://projects.tcag.ca/variation/ are helping to establish AGH as the primary clinical tool for recognition of genomic imbalance that causes ID and other birth defects [33,34,37,41,67-70]. It seems likely, however, that no perfect AGH platform for detection of pathogenic CNVs may ever exist and that effective clinical interpretation of these studies will continue to require considerable skill and experience [33,50,71].

## Methods

### Patients and Families

We studied 100 children with idiopathic ID who had not been studied by AGH before ("the new cohort") and 54 of the idiopathic ID patients whom we had previously tested with 100 K Affymetrix GeneChip^® ^AGH ("the 100 K cohort"). Ten of the 54 patients in the 100 K cohort had previously been found to have pathogenic genomic imbalance; the other 44 patients had previously been reported to have normal 100 K GeneChip^® ^AGH (see Additional File 1: Supplemental Table S1) [10,16]. We also performed 500 K AGH on both unaffected parents of each child.

All of the children were assessed by a clinical geneticist who was unable to determine the cause of the ID despite thorough clinical evaluation and testing that included routine karyotyping with at least 450-band resolution. Subjects were selected for AGH testing because they had ID or developmental delay and at least one of the following additional clinical features: one major malformation, microcephaly, abnormal growth, or multiple minor anomalies. Informed consent was obtained from each family, and assent was also obtained from the child, if possible. The study was approved by the University of British Columbia Clinical Research Ethics Board.

### DNA Preparation

DNA was extracted from whole blood with a Gentra Puregene DNA Purification Kit (Qiagen, Hilden, Germany) according to the manufacturer's instructions. The DNA was precipitated in 70% alcohol, resuspended in hydration solution, and stored at 4°C.

### Hybridization to GeneChip^® ^Mapping 500 K Arrays

DNA degradation, labeling, hybridization, and scanning were performed according to the manufacturer's protocols http://www.affymetrix.com using an Affymetrix Fluidics Station 450, Affymetrix Hybridization Oven 640 and Affymetrix GeneChip Scanner 3000 (Affymetrix, Inc., Santa Clara, CA, USA).

### Copy Number Analysis

Chip-to-chip normalization, standardization to a reference, genotype detection, and copy number estimation on a single SNP basis were performed using the Affymetrix Power Tools (version 1.6.0) software suite http://www.affymetrix.com, as previously described [30]. Estimation of CNV boundary positions was done using Significance of Mean Difference (SMD), a method that we developed. Briefly, the mean of SNP copy number estimates (or log_2 _ratios) within a CNV was compared to the mean of those on the rest of the chromosome, and the probability that the null hypothesis of Student's t-test was true, i.e., that the means were from the same distribution, was calculated. A search using this statistic was conducted over different CNV lengths to find the position and length (in number of SNPs) that yielded the lowest probability, defining the boundaries of a putative CNV. For each sample, a random data set was produced by shuffling the genomic positions of the data, and an identical search was conducted. The results of this search represent false discoveries due to the random variation of the individual SNP data.

**Figure 3:**
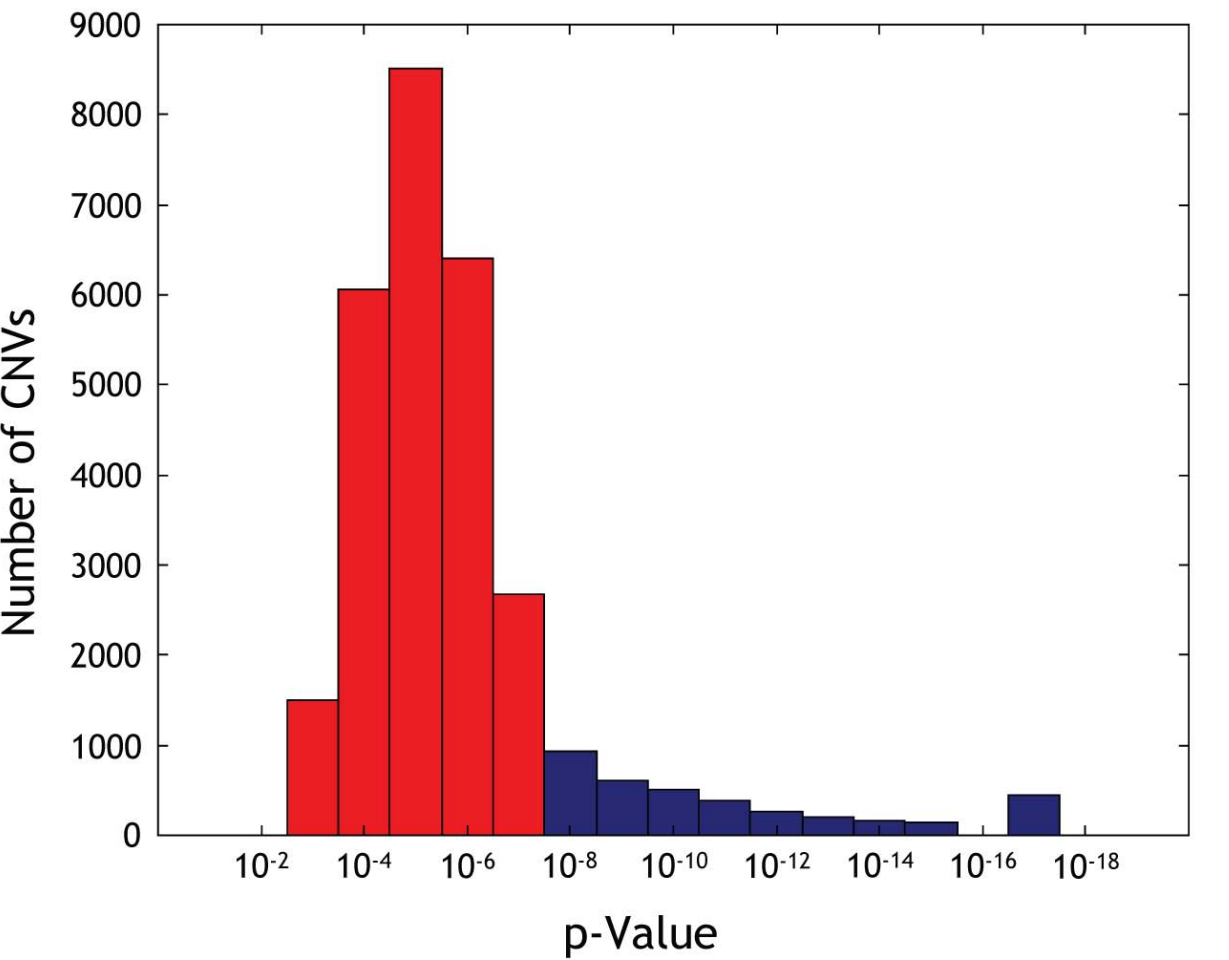
**P-value distribution of apparent CNVs detected by SMD in 462 samples from 154 ID trios**. Data are from the analysis performed by 500 K GeneChip^® ^AGH. Some of these apparent CNVs were merged into larger CNVs at a later stage of the analysis, but most represent individual aberrations. Apparent CNVs with p-values less than 1 × 10^-8 ^were analysed further to determine if they were inherited or had occurred *de novo*. 4577 apparent CNVs were found with p-values below this threshold (shown in blue in the figure). The bin plotted at 10^-17 ^actually contains all CNVs with p < 10^-16^, which is the lower limit of the tables used to calculate p-values.

The p-value distribution of apparent CNVs detected by SMD in all 462 samples (both normal parents and the affected child from 154 ID trios) is shown in Figure 3. We observed that a CNV call with a p-value of 1 × 10^-8 ^usually had a false discovery rate of less than 5%, while a call with a p-value of 1 × 10^-7 ^often had a false discovery rate of 30%, with some variation from sample to sample.

Since SMD compares a putative CNV to the rest of the chromosome, aberrations on the X chromosome are detected equivalently well for males and females. The pseudo-autosomal regions of the X-chromosomes are exceptions, but CNVs there can be detected as a function of the reference set used in the analysis. GeneChip^® ^500 K arrays do not contain Y-chromosome probes.

The SMD search was performed on each child with each parent as a reference. Since our goal was to find duplications and deletions that were *de novo*, a criterion for selection of CNVs for further analysis was that the child have the same aberration with each parent as reference. Correct parental relationships were confirmed in all trios by use of the SNP genotyping calls.

Every putative *de novo *CNV call was evaluated by another analysis conducted using a large reference set of individuals. Early in the study, the 48 sample reference set available from Affymetrix was used, but later a set of 50 mothers from our own data was used. The use of a local reference set significantly reduced noise. The use of the large reference set was required to detect the occurrence of aberrations in both parents that were not inherited by the child. Putative *de novo *CNVs were further evaluated by visual examination of the copy number plots in comparison to both parents as well as of the plots for the child and both parents in comparison to the large reference set.

### Validation of *De Novo *CNVs

Putative *de novo *CNVs identified by 500 K AGH were validated by fluorescence *in situ *hybridization (FISH) or multiplex ligation-dependant probe amplification (MLPA), Agilent^® ^244 K AGH, or repeat cytogenetic analysis. FISH was performed with BAC or fosmid probes selected using the University of California at Santa Cruz Genome Browser [72] and the May 2006 assembly of the human genome sequence. BAC or fosmid DNA was isolated by small-scale (miniprep) preparation and was labeled with Spectrum Red or Green (Vysis, Abbott Molecular, Abbott Park, IL, USA) by use of a Vysis nick translation reagent kit. The labeled product was mixed with 3 mg of human Cot-1 DNA (Invitrogen, Life Technologies Corporation, Carlsbad, CA, USA) and was isolated by means of a standard DNA precipitation method. Cytogenetic pellets were prepared according to standard clinical procedures, and chromosomes and nuclei were visualized by counterstaining with 4',6-diamidino-2-phenylindole. For deletions, at least 10 metaphase cells were analyzed, and interphase nuclei were examined but not counted. For duplications, at least 10 metaphase cells and at least 50 interphase nuclei were analyzed. All FISH probes were tested on metaphase spreads from unaffected individuals to assure proper hybridization.

MLPA was performed using the P070 subtelomeric kit (MRC Holland, Amsterdam, The Netherlands); all positive results were confirmed with another kit (P036B). The procedure was conducted according to the manufacturer's recommendations. Briefly, the patient's DNA was diluted in PCR-grade water and quality was assessed via spectrophotometry (Nanodrop^®^, Thermo Scientific, Wilmington, DE, USA). The hybridization solution (SALSA probe-mix and MLPA buffer) was added to a final DNA concentration of 60 ng/μl. DNA was denatured at 90°C, then hybridized for 16-20 hours at 60°C. Ligation was performed at 54°C for 15 minutes, and the ligated product was denatured at 98°C for 5 minutes and then amplified by PCR (SALSA PCR buffer, PCR-primers and polymerase). The PCR product was run for fragment analysis on an ABI 3130 sequencer (Applied Biosystems, Life Technologies, Carlsbad, CA, USA). Normalization of the data and analysis of the MLPA results were conducted using Coffalyser v3.5 software provided by MRC Holland (Amsterdam, The Netherlands).

AGH was performed with Agilent^® ^244 K oligonucleotide arrays (Agilent Technologies Inc., Santa Clara, CA, USA) according to the manufacturer's instructions. Two arrays were used for each trio, one in which the child's DNA was hybridized against the father's DNA, and another in which the child's DNA was hybridized against the mother's DNA. Captured images were analysed with Feature Extraction v 9.1 and CGH Analytics 3.5.14 (Agilent Technologies Inc., Santa Clara, CA, USA).

Cytogenetic analysis was performed on peripheral blood cultures after preparation and G-banding of metaphase chromosomes using standard clinical methods.

### Uniparental Disomy (UPD)

Given the genotypes for the child, mother, and father, errors in mendelian transmission were identified and their frequency compared to normal or technical error rates, which were very low. Essentially, the occurrences of an AA parent with a BB child, or vice versa, were counted. Graphical tools were developed to distinguish heterodisomy and isodisomy by visualization and to determine the parent of origin [30].

Confirmation of uniparental disomy was obtained by genotyping microsatellite markers chosen for high heterozygosity values. The following markers were genotyped and were informative for chromosome 11: D11S1363 (AFMA134WH5), D11S4046 (AFMB042YF5), D11S1318 (AFM218XE1), D11S4088 (AFMA155TE9) and D11S4146 (AFMB072WE5). The following markers were genotyped and were informative for chromosome 16: D16S3093 (AFMB308ZH9), D16S409 (AFM161XA1), D16S515 (AFM340YE5) and D16S402 (AFM031XA5). 25 ng of DNA diluted in 10:1 TE buffer were amplified for each PCR reaction. Primers fluorescently labeled with either 6-fam or HEX (Applied Biosystems, Life Technologies, Carlsbad, CA, USA) were used in conjunction with AmpliTaq Gold^® ^PCR kit reagents (Applied Biosystems, Life Technologies, Carlsbad, CA, USA). PCR was performed using the following steps: 95°C for 10 minutes; 30 cycles of 95°C for 30 seconds, 55°C for 30 seconds, and 72°C for 90 seconds; and a final step at 72°C for 7 minutes. The resulting PCR product in a volume of 1 μl was combined with 9 μl formamide (Applied Biosystems, Life Technologies, Carlsbad, CA, USA) and 0.3 ul GeneScan ROX 500 (Applied Biosystems, Life Technologies, Carlsbad, CA, USA), denatured at 95°C for 5 minutes, quickly chilled in ice and loaded onto a Genetic Analyzer 3130XL (Applied Biosystems, Life Technologies, Carlsbad, CA, USA). Data were visualized using GeneMapper v4.0 (Applied Biosystems, Life Technologies, Carlsbad, CA, USA).

## List of Abbreviations Used

AGH: array genomic hybridization; BAC: bacterial artificial chromosome; bp: base pairs; CNV: copy number variant; del: deletion; FISH: fluorescence *in situ *hybridization; ID: intellectual disability; K: thousand; Mb: megabase; MLPA: multiplex ligation-dependant probe amplification; PCR: polymerase chain reaction; SMD: significance of mean difference; SNP: single nucleotide polymorphism; UPD: uniparental disomy.

## Authors' contributions

JMF and MAM conceived, designed and coordinated this study. PB developed and implemented the consent process, and JMF, SA, LA, LA, PB, CB, WTG, SL, EL, PM, JLM, BCM, MSP, GAR, MIVA and S-LY recruited patients. Clinical evaluations were performed by LA, LA, CB, JMF, WTG, SL, EL, PM, BCM, MSP, MIVA and S-LY. AB and SC performed the array genomic hybridizations, and AB, ADD, PE, SF, SL, HIL, HQ, MAM and JMF interpreted the AGH data. Confirmatory FISH and MLPA studies were done by PE, DC, EL and JM. LA, LA, CV, PE, WTG, SL, EL, PM, JLM, BCM, MSP, MIVA, S-LY, FRZ and JMF performed the genotype-phenotype correlations. JMF drafted the manuscript, which was critically reviewed and edited by AB, ADD, PE, SL, JLM, MSP, FRZ and MAM. All authors read and approved the final manuscript.

## Supplementary Material

Additional file 1 **Supplemental Table S1**. 54 ID trios from the 100 K cohort on whom 500 K GeneChip^® ^AGH was performed. The findings in families in whom *de novo *CNVs were found are summarized in Table 3. Click here for file

Additional file 2 **Supplemental Figure S1. Size distribution of pathogenic CNVs detected by 100 K and 500 K AGH in idiopathic ID trios**. The number of deletions (red bars) or duplications (blue bars) in each size class is shown for 8 submicroscopic deletions and 2 submicroscopic duplications found in 100 idiopathic ID trios studied with 100 K AGH (empty and stippled bars) and for 22 submicroscopic deletions and 5 submicroscopic duplications found in 154 idiopathic ID trios studied with 500 K AGH (filled bars and stippled bars). 46 trios in the 100 K cohort were studied only with 100 K GeneChip^® ^AGH (empty bars), 54 other trios from the 100 K cohort were studied with both 100 K and 500 K GeneChip^® ^AGH (stippled bars), and 100 additional trios constituted a new cohort who were studied only with 500 K GeneChip^® ^AGH (solid bars). Data for the 100 K trios are from [10]. The 500 K data include two unbalanced reciprocal translocations, for each of which both a deletion and a duplication are shown in the figure. Click here for file
