# Supplementary material for: Detection of pathogenic copy number variants in children with idiopathic intellectual disability using 500 K SNP array genomic hybridization
Source: BMC Genomics. 2009 Nov 16;10:526. doi: 10.1186/1471-2164-10-526 (PMC2781027; doi:10.1186/1471-2164-10-526)
Supplement: Additional file 1 — Supplemental Table S1. 54 ID trios from the 100 K cohort on whom 500 K GeneChip® AGH was performed. The findings in families in whom de novo CNVs were found are summarized in Table 3. [file 1471-2164-10-526-S1.DOC]

**Supplemental Table S1.**  **54 ID trios from the 100K cohort on whom 500K GeneChip® AGH was performed.** The findings in families in whom *de novo* CNVs were found are summarized in Table 3.

| **Family** | **Affymetrix 100K AGH Result** | **Affymetrix 500K AGH Result** |
| --- | --- | --- |
| 134 | Normal | Normal |
| 167 | Normal | Normal |
| 392 | Normal | Normal |
| 395 | Normal | Normal |
| 421 | Normal | Normal |
| 451 | Normal | Normal |
| 491 | Normal | Normal |
| 517 | Normal | Normal |
| 621 | Normal | Normal |
| 648 | Normal | Normal |
| 674 | Normal | Normal |
| 1234 | Normal | Normal |
| 1252 | Normal | Normal |
| 1280 | Normal | Normal |
| 1523 | Normal | Normal |
| 1895 | del(13)(q12.11q12.13) | del(13)(q12.11q12.12) |
| 1978 | Normal | Normal |
| 2135 | Normal | Normal |
| 3153 | Normal | Normal |
| 3476 | del(4)(q21.21q22.1) | del(4)(q21.21q22.1) |
| 3553 | Normal | Normal |
| 3595 | Normal | Normal |
| 3717 | Normal | Normal |
| 3890 | Normal | dup(8)(q23.2q23.3) |
| 3923 | Normal | Normal |
| 4268 | Normal | Normal |
| 4357 | Normal | Normal |
| 4462 | Normal | Normal |
| 4535 | Normal | Normal |
| 4794 | dup(16)(p13.3p13.3) | dup(16)(p13.3p13.3) |
| 4818 | del(12)(q14.2q15) | del(12)(q14.2q15) |
| 4840 | Normal | del(4)(p16.3p16.3) |
| 5003 | del(2)(p16.3p16.3) | del(2)(p16.3p16.3) |
| 5202 | Normal | Normal |
| 5495 | Normal | Normal |
| 5566 | del(14)(q11.2q11.2) | del(14)(q11.2q11.2) |
| 5994 | mosaic trisomy 9 | mosaic trisomy 9 |
| 6128 | Normal | Normal |
| 6349 | Normal | Normal |
| 6545 | del(7)(p22.1p22.2) | del(7)(p22.1p22.2) |
| 6607 | Normal | Normal |
| 6789 | Normal | Normal |
| 7551 | Normal | Normal |
| 7690 | Normal | Normal |
| 7807 | del(22)(q12.1q12.1) | del(22)(q12.1q12.1) |
| 8005 | Normal | Normal |
| 8305 | Normal | Normal |
| 8326 | del(14)(q11.2 q11.2) | del(14)(q11.2 q11.2) |
| 8541 | Normal | Normal |
| 8552 | Normal | Normal |
| 8785 | Normal | Normal |
| 8939 | Normal | Normal |
| 9299 | Normal | Normal |
| 9923 | Normal | Normal |
