# Supplementary figures and images for: Detection of pathogenic copy number variants in children with idiopathic intellectual disability using 500 K SNP array genomic hybridization
Source: BMC Genomics. 2009 Nov 16;10:526. doi: 10.1186/1471-2164-10-526 (PMC2781027; doi:10.1186/1471-2164-10-526)

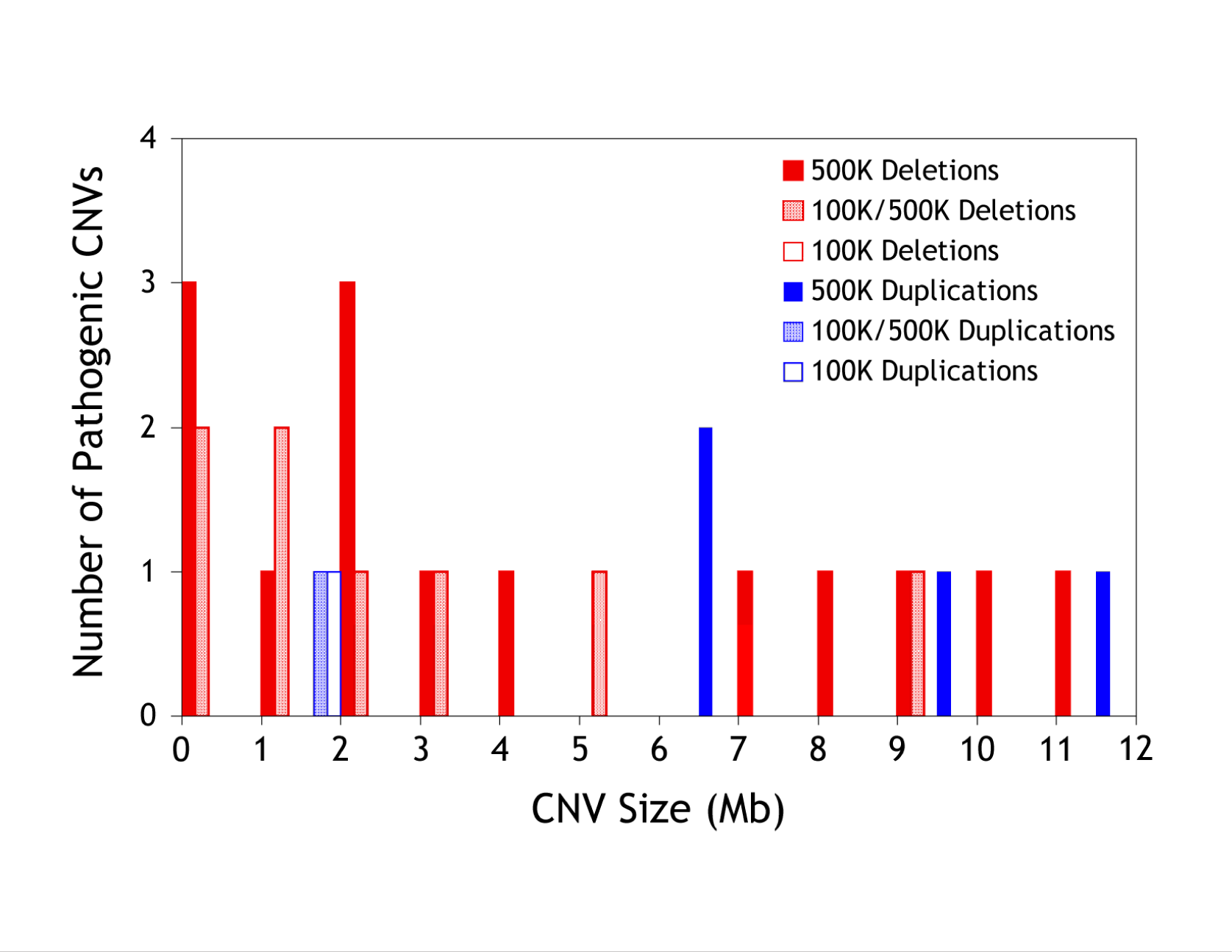

Supplement: Additional file 2 — Supplemental Figure S1. Size distribution of pathogenic CNVs detected by 100 K and 500 K AGH in idiopathic ID trios. The number of deletions (red bars) or duplications (blue bars) in each size class is shown for 8 submicroscopic deletions and 2 submicroscopic duplications found in 100 idiopathic ID trios studied with 100 K AGH (empty and stippled bars) and for 22 submicroscopic deletions and 5 submicroscopic duplications found in 154 idiopathic ID trios studied with 500 K AGH (filled bars and stippled bars). 46 trios in the 100 K cohort were studied only with 100 K GeneChip® AGH (empty bars), 54 other trios from the 100 K cohort were studied with both 100 K and 500 K GeneChip® AGH (stippled bars), and 100 additional trios constituted a new cohort who were studied only with 500 K GeneChip® AGH (solid bars). Data for the 100 K trios are from [10]. The 500 K data include two unbalanced reciprocal translocations, for each of which both a deletion and a duplication are shown in the figure. [file 1471-2164-10-526-S2.PNG]
